# Impact of Spinal Anesthesia Dosage in Elective Cesarean Section on the Duration of Stay in Post-Anesthesia Care Unit at the Women’s Health Hospital, National Guard Health Affairs

**DOI:** 10.7759/cureus.75626

**Published:** 2024-12-13

**Authors:** Amer A Alkinani, Badar Albabtean, Hamad Alfaris, Abdulmalik Alarwan, Abdullah Al Harbi, Mohammed Alrajeh, Talal Alhumaid, Abdullah Alhobabi, Faisal T Alanazi, Raed Alzahrani, Naif Alsaber

**Affiliations:** 1 Anesthesia, King Abdulaziz Medical City Riyadh, Riyadh, SAU; 2 Anesthesiology, King Abdulaziz Medical City Riyadh, Riyadh, SAU; 3 College of Medicine, King Saud Bin Abdulaziz University for Health Sciences, Riyadh, SAU; 4 College of Medicine, King Saud Bin Abdulaziz University for Health Sciences, riyadh, SAU; 5 Medicine, King Khalid University Hospital, Riyadh, SAU

**Keywords:** bupivacaine, caesarean section, duration of stay, marcaine, pacu

## Abstract

In the field of obstetrics, cesarean sections have now become the most prominent procedure for the delivery of newborns. Cesarean sections may be handled with a variety of different anesthetic approaches, of which most focus seems to be on that of spinal forms, due to their rapid and effective action. Dosages of spinal anesthesia formulations differ depending on multiple variables, such as depth of anesthesia, level of analgesia, and desired duration of effects. Furthermore, length of stay in the hospital is also a crucial variable to take into account when using spinal anesthesia due to its implications for patient comfort, quality of care, and return to daily life. Hyperbaric bupivacaine is a mainstay agent in regard to cesarean section anesthesia, with the most commonly used dosages being 1.8 mL and 2.0 mL. This study aims to assess the difference in duration of stay in the post-anesthesia care unit between patients receiving 1.8 mL and 2.0 mL of 0.5% hyperbaric bupivacaine. Of the 306 patients who underwent elective cesarean sections, 63 patients received 2.0 mL of 0.5% hyperbaric bupivacaine and 243 patients received 1.8 mL of 0.5% hyperbaric bupivacaine.

## Introduction

Cesarean section is now the most common procedure performed in North America and is on a rapid increase in the rest of the world as well [1,2]. In Saudi Arabia, according to the most recent update to the country’s health activities analysis by the Ministry of Health, cesarean sections account for 35.7% of all deliveries in the country [3]. Importantly, the form of anesthesia employed is significant to both the protection of the patient and the newborn and pain management in patients undergoing the procedure [4]. Types of anesthesia used in cesarean sections worldwide most prominently include general anesthesia (GA), spinal anesthesia, epidural anesthesia, and combined forms of the aforementioned types [5]. In Saudi Arabia, numerous studies have been published regarding the preferred option for anesthesia by women undergoing the procedure electively, with the overwhelming majority favoring spinal anesthesia [6].

Beyond patient preference, spinal anesthesia has been illustrated to be favored overall over GA due to GA’s association with complications such as aspiration, airway risks, intraoperative awareness, and an increase in risk of blood loss due to uterine atony, as seen with the use of halothane for example [7]. Furthermore, compared with GA, spinal anesthesia has been associated with an overwhelming reduction in maternal and fetal mortality and morbidity. For the last few decades, spinal anesthesia, as a form of neuroaxial anesthesia, has been considered the gold standard for women undergoing elective cesarean section. This is due to spinal anesthesia's ability to provide a more dense sensory block than that provided with an epidural, while also maintaining the advantage of a lower risk of local anesthetic toxicity compared to epidural anesthesia [8]. Of the spinal anesthesia formulations used in cesarean sections, hyperbaric bupivacaine is the most predominantly used. Hyperbaric bupivacaine, commonly known by its market name “Heavy Marcaine,” is the most commonly used anesthetic in cesarean sections due to its ease of use, inexpensive cost, and rapid predictable action providing analgesia and muscle relaxation [9,10].

Hyperbaric bupivacaine has become a core staple in obstetrical anesthesia, particularly elective cesarean sections. Acting as a powerful local anesthetic amide agent, bupivacaine is administered directly into the cerebrospinal fluid to produce anesthesia in cesarean section patients. The agent’s anesthetic action is mediated through its blockade of sodium channels on neuronal membranes. Through its blockade of sodium channels, the agent provides significant hindering to both the depolarization of nerve membranes and the propagation of impulses along affected nerves, allowing for effective motor and sensory blockade [11]. Bupivacaine in particular provides extensive motor block with short onset and longer duration, as opposed to other local anesthetics [12]. Bupivacaine has been illustrated to have varying effects on hemodynamic control, analgesia, and post-operative nausea and vomiting depending on the dosage administered [13,14]

While bupivacaine has proven itself to be a remarkable agent in the obstetrical anesthesia setting, the anesthetic variation of effect based on dosage has yet to be fully illustrated. A study published in 2023 described the variation of effect between differing doses and noted a statistically significant increase in duration of motor block and length of stay in the post-anesthesia care unit (PACU) in patients who received a relatively higher dose of bupivacaine as opposed to a lower dose [15]. Moreover, another study published in the International Journal of Medical Anesthesiology noted that patients who received a higher fixed dosage of bupivacaine were more likely to undergo motor block faster, although at higher risk of hypotension, bradycardia, and prolongation of stay in the PACU, as opposed to patients who received lower dosages for their elective cesarean sections [16]. There remains, however, a scarcity of articles that explore the variation in the length of stay between differing dosages of bupivacaine for spinal anesthesia in elective cesarean sections, and even more rare is a study exploring the phenomenon among Saudi Arabian patients. The aim of this study is to fully illustrate and compare the effects of bupivacaine at 1.8 mL as opposed to 2.0 mL on the length of stay of post-operative cesarean sections in the PACU.

## Materials and methods

Study design

This is a retrospective cohort study that included patients who underwent cesarean section, as documented through electronic health records from October 1, 2023, to September 30, 2024, at Woman Health Hospital (WHH), Riyadh, Kingdom of Saudi Arabia. The BESTCare system was utilized to capture all cesarean section cases, providing full access to each patient’s comprehensive medical record.

Selection criteria

In this study, patients who received spinal anesthesia due to elective cesarean section and medically free without any comorbidities, had body mass index (BMI) < 35 kg/m^2^, had documented hyperbaric bupivacaine use, and were classified as American Society of Anesthesiology (ASA) class 2 were included. Patients with a history of abdominal surgery/trauma and those with height less than 150 cm or greater than 170 cm were excluded from the study. These exclusion settings have been set to ensure large variation in patients excluded and avoid the skewing off data to reduce confounding variables that may affect assessment of duration variation as well as analgesia requirements in patients due to the sensitive properties of the anesthetic. Furthermore, to avoid confounding of our results, no adjuvant agents were used alongside spinal anesthetic, such as fentanyl, morphine, or dexmedetomidine.

Sample size

**Figure 1:**
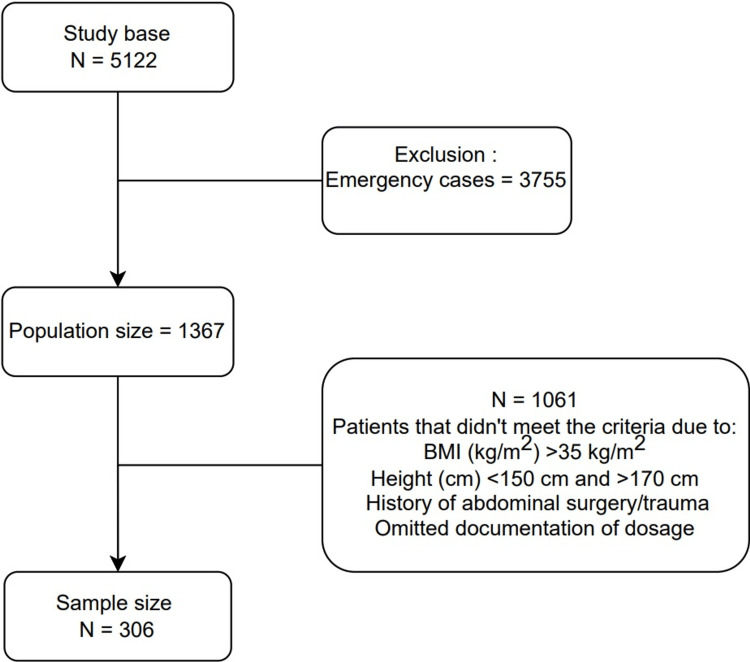
Inclusion and exclusion of patients to form the study sample

A total of 1,367 women underwent cesarean section at WHH from October 2023 to October 2024. After meeting the inclusion and exclusion criteria, our calculated sample size was 306 patients (Figure 1).

Data collection

The study group identified and reviewed the complete medical records of all patients who met the inclusion criteria using a standardized data abstraction form. The patients were divided into two subgroups: one subgroup received 1.8 mL of bupivacaine and the other received 2.0 mL of bupivacaine. For each patient, the duration of surgery and length of stay in the PACU were recorded, along with any complications experienced during the PACU stay. The recorded complications included pain, itchiness, nausea, and vomiting, along with the medications administered to manage these complications during the PACU period.

Statistical analysis

SPSS Version 27 (IBM Corp., Armonk, NY) was used to analyze the findings of this study. Our continuous variables satisfied the rules of Gaussian distribution; as such, t-test was used to compare the means of different groups. Chi-square was used to compare the categorical variables excepted by events that necessitated the use of Fisher’s exact test for smaller products of observed and expected values.

## Results

Table 1 compares the baseline characteristics of patients receiving either 1.8 mL or 2.0 mL of Marcaine. There was no significant difference in age between the two groups (p = 0.602). However, the BMI was significantly lower in the 1.8 mL group compared to the 2.0 mL group (p < 0.001).

**Table 1:** Comparison of baseline characteristics Characteristics of both dosage groups were compared using the independent sample t-test to assess for baseline homogeneity. Statistical significance is achieved if p-value is <0.05.

Variable	Mean ± SD (1.8 mL) (n= 243)	Mean ± SD (2.0 mL) (n= 63)	p-Value
Age (years)	31.5 (±5.3)	33.5 (± 5.5)	0.602
BMI (kg/m^2)^	27.7 (±3.5)	29.6 (±3.5)	<0.001

In Table 2, the incidence of adverse effects is illustrated among patients in terms of frequency. The incidence of nausea and vomiting was similar across both groups (p = 0.471). However, the occurrence of pain was significantly higher in the 1.8 mL group (p = 0.014). The types of analgesics used, including acetaminophen, NSAIDs, and opioids, did not significantly differ between the groups (p = 0.911). Itchiness did not differ significantly across the two groups (p = 0.425).

**Table 2:** Effects of 1.8 mL versus 2.0 mL on clinical parameters Categorical outcomes of both dosage groups were compared using the chi-square and Fisher's exact tests. Statistical significance is achieved if p-value is <0.05.

Dose of Marcaine	Frequency (1.8 mL) (n= 243)	Frequency (2.0 mL) (n= 63)	p-Value
Nausea and vomiting	11	1	0.471
Pain	40	19	0.014
Analgesia use	Acetaminophen: 22	Acetaminophen: 9	0.911
	NSAIDs: 10	NSAIDs: 5	
	Opioids: 6	Opioids: 2	
Itchiness	16	3	0.425

Table 3 elucidates the differences in duration of surgery and length of stay between both the 1.8 mL and 2.0 mL study groups. The duration of surgery tended to be slightly longer in the 2.0 mL group at nearly significant values (p = 0.051). Conversely, the length of stay in the PACU was nearly identical between groups (p = 0.999), indicating no difference in immediate postoperative recovery times based on the dose administered.

**Table 3:** Effects of 1.8 mL versus 2.0 mL on the duration of surgery and PACU stay Clinical outcomes of both dosage groups were compared using the independent sample t-test. Statistical significance is achieved if p-value is <0.05. PACU, post-anesthesia care unit

Dose of Marcaine	Mean ± SD (1.8 mL) (n= 243)	Mean (2.0 mL) (n= 63)	p-Value
Duration of surgery (min)	69.5 (±22.1)	75.8 (±23.8)	0.051
Length of Stay in PACU (min)	93.7 (±35.0)	93.7 (±34.4)	0.999

## Discussion

This study examined the impact of spinal anesthesia dosage, specifically 1.8 mL versus 2.0 mL of hyperbaric 0.5% bupivacaine, on the length of stay in the PACU in patients undergoing elective cesarean sections. No significant difference in PACU stay duration was observed between the two dosage groups (p = 0.999), suggesting that a minor increase in Marcaine dose from 1.8 mL to 2.0 mL does not meaningfully alter this population's immediate postoperative recovery time. This finding is consistent with research indicating that moderate increases in spinal anesthetic dosage may not necessarily prolong PACU recovery time [15]. This underscores the potential for individualized dosing tailored to patient needs without negatively impacting overall PACU resource utilization. One notable result is the significantly higher incidence of postoperative pain in the 1.8 mL group compared to the 2.0 mL group (p = 0.014). This suggests that a higher dose of Marcaine may enhance postoperative pain relief. Lower dosages could result in shorter or less intense motor and sensory blockades, potentially increasing postoperative discomfort. Previous research indicates that while lower spinal anesthetic dosages may reduce side effects, they might compromise pain management [17]. Thus, a dosage of 2.0 ml could effectively manage postoperative pain without significantly extending PACU stay Regarding adverse effects, the rates of postoperative nausea and vomiting (p = 0.471) and itching (p = 0.425) did not show a significant difference between the two dosage groups. Studies indicate that the dosage of Marcaine (bupivacaine) has a negligible effect on adverse effects; instead, it is the individual patient characteristics and simultaneous medications that play a more significant role [18]. These results align with other research findings indicating that outcomes for postoperative nausea and vomiting are often more heavily influenced by factors such as hypotension or opioid use than by the specific dosage of bupivacaine given. The data also show a trend toward a slightly longer duration of surgery in the 2.0 mL group (p = 0.051). Although the trend is approaching significance, it may indicate that a higher dosage of Marcaine provides more stable anesthesia, allowing surgeons additional time if deemed necessary without needing any supplementary anesthesia. Research on bupivacaine dosages has shown that higher doses can lead to more stable nerve blocks, which could be advantageous during longer procedures [18]. Nonetheless, caution should be exercised when interpreting this finding due to its near-significance and the possibility that it may be incidental rather than clinically meaningful.

Clinicians might decide on the Marcaine dosage based on other considerations, such as desired pain management, rather than concerns about assigning PACU resources, as seen by the absence of variation in PACU stay duration. The 2.0 mL dosage might be the best option for optimizing postoperative comfort in cesarean patients because it seems to provide better pain management without exacerbating side effects such as nausea and itching. However, when planning anesthetic management, the 2.0 mL dose's potential for somewhat longer surgical periods should be taken into account.

Study limitations

This research has a number of limitations. Firstly, there was a remarkable difference in the number of patients in both groups, with 243 patients in the 1.8 mL group compared to 63 in the 2.0 mL group. Moreover, due to the manual recording of variables such as pain, nausea, vomiting, itchiness, and duration of stay within the PACU, it is subjected to the possibility of human communication error. Furthermore, intraoperative hemodynamics and need for vasopressors were not recorded or analyzed, which may have also provided another area of difference between the two forms of administration. Finally, being a single-center study, the outcomes may not be entirely applicable to other environments where patient demographics, anesthetic methods, and PACU practices differ.

## Conclusions

In conclusion, the findings indicate that increasing the heavy Marcaine dosage from 1.8 mL to 2.0 mL does not impact the duration of stay in the PACU. Additionally, a higher dosage may offer more effective pain management after surgery without causing extra side effects. These insights contribute to the ongoing discussion regarding optimal spinal anesthesia dosing strategies for cesarean sections and could help guide more personalized anesthesia care, enhancing postoperative comfort while maintaining PACU efficiency.
